# Oxalotrophy, a widespread trait of plant-associated *Burkholderia* species, is involved in successful root colonization of lupin and maize by *Burkholderia phytofirmans*

**DOI:** 10.3389/fmicb.2013.00421

**Published:** 2014-01-09

**Authors:** Thomas Kost, Nejc Stopnisek, Kirsty Agnoli, Leo Eberl, Laure Weisskopf

**Affiliations:** ^1^Laboratory of Microbiology, Institute of Plant Biology, University of ZurichZurich, Switzerland; ^2^Ecology of Noxious and Beneficial Organisms, Institute of Sustainability SciencesAgroscope, Zurich, Switzerland

**Keywords:** oxalate, root colonization, *Burkholderia*, PGPR, oxalate decarboxylase

## Abstract

Plant roots and shoots harbor complex bacterial communities. Early seed and plantlet colonization plays a key role in determining which bacterial populations will successfully invade plant tissues, yet the mechanisms enabling plants to select for beneficial rather than harmful populations are largely unknown. In this study, we demonstrate a role of oxalate as a determinant in this selection process, using members of the genus *Burkholderia* as model organisms. Oxalotrophy, i.e., the ability to use oxalate as a carbon source, was found to be a property strictly associated with plant-beneficial species of the *Burkholderia* genus, while plant pathogenic (*B. glumae, B. plantarii*) or human opportunistic pathogens (*Burkholderia cepacia* complex strains) were unable to degrade oxalate. We further show that oxalotrophy is required for successful plant colonization by the broad host endophyte *Burkholderia phytofirmans* PsJN: an engineered Δ*oxc* mutant, which lost the ability to grow on oxalate, was significantly impaired in early colonization of both lupin and maize compared with the wild-type. This work suggests that in addition to the role of oxalate in heavy metal tolerance of plants and in virulence of phytopathogenic fungi, it is also involved in specifically recruiting plant-beneficial members from complex bacterial communities.

## Introduction

In the rhizosphere, most bacteria rely on root exudates as a source of carbon and energy. Exudates are of highly diverse chemical nature, from small carboxylates to complex phenolic compounds, and their secretion depends mostly on plant species and growth conditions. In nutrient-limited as well as in heavy-metal contaminated soils, exudation of organic acids is increased. This differential exudation of specific compounds has been shown to influence bacterial community structure (Weisskopf et al., 2005, 2008; Badri et al., 2009; Doornbos et al., 2012; Chaparro et al., 2013). Carboxylates such as citrate and malate are a major source of carbon for rhizosphere bacteria, and malate has even been postulated to act as a signal to recruit beneficial microorganisms (Rudrappa et al., 2008). In contrast, using oxalate as carbon source, a phenotype referred to as “oxalotrophy,” is a rare trait of bacteria, although it occurs across a wide range of phylogenetically distant groups (Sahin, 2003; Khammar et al., 2009). In addition to citrate and malate, which are common components of root exudates, oxalate has also been shown to be a major root exudate of soil-grown plants (Dessureault-Rompre et al., 2007). However, neither the function of oxalate in recruiting specific microbes nor the relevance of oxalotrophy for bacterial rhizosphere competence has so far been investigated.

Members of the *Burkholderia* genus are frequently retrieved in plant microbiome surveys and seem to play a substantial role in direct plant growth promotion or in protection against soil-borne fungi (Mendes et al., 2007; Opelt et al., 2007; Compant et al., 2008; Li et al., 2008; Hardoim et al., 2011; Ikeda et al., 2013). Yet, beside plant beneficial members of the genus (e.g., *B. phytofirmans, B. phymatum*), others represent a threat to human health, such as the opportunistic pathogens of the *Burkholderia cepacia* complex (Mahenthiralingam et al., 2005). In an effort to characterize the bacterial communities living in and on the roots of white lupin, we have recently shown by both culture-independent and culture-dependent approaches that *Burkholderia* species are predominant members of the bacterial community inhabiting the cluster roots (Weisskopf et al., 2011). In addition to their ability to grow on citrate or malate, almost all isolated *Burkholderia* strains were able to use plant-secreted oxalate as a carbon source: 98% of the *Burkholderia* strains were oxalotrophic, compared with only 2% of the non *Burkholderia* strains isolated from the same environment. Moreover, *Burkholderia* sequences and strains almost exclusively belonged to the plant beneficial species and not to the opportunistic pathogenic ones (Weisskopf et al., 2011). These results led us to hypothesize that the capacity to utilize plant-exuded oxalate might explain why the roots of white lupin are strongly enriched for *Burkholderia* species. To test this hypothesis, we determined the capacity to utilize oxalate among a wide range of *Burkholderia* strains that belong either to plant beneficial or to opportunistic pathogenic species. In addition, we mutated the oxalotrophy pathway in the plant beneficial endophytic *B. phytofirmans* and monitored seed and root colonization of the mutant and the wild-type strains in white lupin and in maize.

## Materials and methods

### Strains, plasmids and culture media

Strains and plasmids used in this study are listed in Table S1. For long-term storage, bacterial strains were kept at −80°C in 50% glycerol. Chemicals were purchased from Sigma Aldrich if not specified otherwise. Bacteria were routinely grown on Luria-Bertani (LB) medium (20 g LB powder (Difco) per liter) and 18 g agar, *Pseudomonas* Isolation Agar (PIA) medium (45 g *Pseudomonas* Isolation Agar (Difco), 5 g additional agar, 20 ml glycerol per liter), or Mueller-Hinton agar (21 g Mueller Hinton Broth (Difco) and 15 g agar per liter). For oxalate degradation assay, AB minimal medium was used with (per liter) 2 g (NH_4_)_2_SO4, 6 g Na_2_HPO_4_, 6 g Na_2_HPO_4_, 3 g NaCl, 2 mM MgCl_2_ × 6 H_2_O, 100 μM CaCl_2_ × 6 H_2_O, 3 μM FeCl_3_ × H_2_O and 40 μl oligoelement solution (10 mg ZnSO_4_ × 7 H_2_O, 13 mg MnCl_2_ × 4 H_2_O, 3 mg Na_2_MoO_4_ × 2 H_2_O, 30 mg H_3_Bo_3_, 20 mg CoCl_2_ × 6 H_2_O, 1 mg CuCl_2_ × H_2_O, 2 mg NiCl_2_ × 6 H_2_O). pH was adjusted to 7. This medium, supplemented with 18 g agar per liter, was used as the first layer of the oxalate degradation medium. A second layer, which contained 7 g calcium oxalate × H_2_O and 12 g agar per liter was freshly stirred and added on the first layer. MS medium contained 2.2 g Murashige and Skoog medium (Sigma-Aldrich) and 5 g agar per liter. pH was adjusted to 5.7 prior to autoclaving.

### Oxalate degradation assay

Strains were grown overnight in 5 ml of AB minimal medium with 5 g l^−1^ glucose as carbon source. 2 ml of the overnight culture were centrifuged at 4000 rpm for 5 min and the pellet was washed twice and resuspended in 1 ml 0.9% NaCl solution. OD_600_ was measured and all samples were diluted with 0.9% NaCl to OD_600_ of 0.2. 50 μl of diluted cell suspension were pipetted onto the double layer oxalate-medium and incubated for at least 2 days at 30°C. The formation of a transparent halo revealed the ability to degrade oxalate.

### Construction of a mutant impaired in oxalate degradation and fluorescent tagging

In *B. phytofirmans* PsJN, the oxalate degradation cluster is located on chromosome 2 and consisted of three genes encoding (i) the oxalate/formate antiporter (Bphyt_6739), (ii) the oxalate decarboxylase (*oxc*, Bphyt_6740), and the formyl-CoA transferase (*frc*, Bphyt_6741) (Weilharter et al., 2011). Unlike the antiporter and the formyl-CoA transferase, the oxalate decarboxylase was present as single copy in the genome, and was thus chosen as a target for mutagenesis. A 1650 bp region spanning Bphyt_6740 (*oxc*) was amplified using XhoI and BglII restriction site-containing primers 5′-GCGCCTCGAGCTGAACGACATCAAAACCAT-3′ and 5′-GCGCAGATCTGATTACTTTTTCATTGCCGC-3′, which were designed using the CLC workbench software and purchased from Microsynth, Balgach, Switzerland. The PCR reaction was performed as follows: 1 cycle of 2 min at 95°C followed by 30 cycles of 30 s at 94°C, 30 s at 48°C, and 100 s at 72°C, and a final extension at 72°C for 5 min. The resulting amplicon was purified using Qiagen PCR purification kit, digested with BglII and XhoI and ligated overnight at room temperature with the vector pSHAFT2 (4552 bp) previously digested with the same enzymes. The ligation product was transformed into *E. coli* CC118λpir cells followed by selection for chloramphenicol resistant clones on LB plates. The resulting plasmid (pSHAFT2 carrying *oxc*) was then isolated and digested with NcoI, a restriction site located in the middle of *oxc*, dephosphorylated and purified. In parallel a trimethoprim resistance cassette was amplified by PCR using NcoI containing primers 5′-GCGCCCATGGCAGTTGACATAAGCCTGTTC-3′ and 5′-GCGCCCATGGTTAGGCCACACGTTCAAGTG-3′, which were designed using the CLC workbench software and purchased from Microsynth, Balgach, Switzerland. The PCR reaction was performed by 1 cycle of 2 min at 95°C followed by 30 cycles of 30 s at 95°C, 30 s at 50°C, and 100 s at 72°C, and a final extension at 72°C for 5 min. The resulting amplicon was digested with NcoI and purified. Ligation was performed overnight and the ligation product was transformed into CC118λ pir cells. Clones were selected on Mueller-Hinton plates supplemented with trimethoprim and correct insertion in the isolated plasmids was verified by restriction with NcoI or XhoI and BglII. This strain carrying the interrupted *oxc* gene was used as donor strain for triparental mating with *E. coli* MM294 strain as a helper and *B. phytofirmans* PsJN as a recipient. 2 ml of overnight culture (5 ml LB medium with appropriate antibiotic) was centrifuged and washed twice in 0.9% NaCl solution. Then the cells were resuspended in 0.5 ml LB media. 100 μl of the helper culture; 100 μl of the donor strain culture were mixed and kept at room temperature (RT) for 20 min and then 100 μl of recipient strain were added. Afterwards 150 μl of the mixed culture were pipetted in drops of about 50 μl on a LB plate and incubated for 6 h at 30°C. Then the cells were harvested, resuspended in 1.5 ml 0.9% NaCl solution, diluted and spread on PIA plates supplemented with trimethoprim. Loss of chloramphenicol resistance was used to select clones where double crossing-over recombination had occurred (see Figure S1 for a diagram of the cloning procedure). Fluorescent tagging of *B. phytofirmans* wild-type and Δ*oxc* mutant was carried out by triparental mating as described above. The donor strains were *E. coli* carrying either the plasmid pBBR1MCS-2-gfpmut3 (GFP, kanamycin resistance) or the plasmid pIN62 carrying the dsRED encoding gene and a chloramphenicol resistance cassette (see Table S1). Transformants were selected on PIA plates with kanamycin or chloramphenicol.

### Plant colonization experiments

#### Early colonization

Two plant species were used as models for the colonization assays of *B. phytofirmans* wild-type and Δ*oxc*-mutant: white lupin (*Lupinus albus* L., cv. Amiga) and maize (*Zea mays* subsp. *mays*, cv. Birko). Seeds were sterilized by vigorous shaking (200 rpm) in 2.5% NaHClO solution 0.2‰ (v:v) Triton X for 5 min, followed by rinsing twice in sterile water and drying under the sterile bench. Seeds were bacterized with *B. phytofirmans* strains using the following procedure: dsRED- or GFP-tagged derivatives of the wild-type strain and the Δ*oxc* mutant, were grown overnight in LB broth. The dsRED-tagged strains were used for single inoculation experiments due to the higher signal intensity compared to the GFP-tagged cells. Cells were harvested by centrifuging for 5 min at 6000 rpm, washed twice in NaCl 0.9% and resuspended in 20 ml NaCl solution to adjust the OD_600_ to 0.25 (corresponding to approximately 10^7^ cells/ml). For mixed inoculations (GFP-tagged wild-type (wt): dsRED-tagged Δ*oxc*, dsRED-tagged wt: GPF-tagged Δ*oxc*), the two strains were mixed after cell washing in a 1:1 ratio (OD_600_ of 0.125 for each strain). 20 surface-sterilized seeds of maize or 20 seeds of lupin were dipped in 10 ml of the respective bacterial suspension and incubated in a Falcon tube for 1 h at room temperature. Control seeds were incubated in NaCl solution. Thereafter, bacterized seeds were washed in NaCl to remove non-attached cells and sterilely transferred to Petri dishes with 1/2 MS medium. Plates were incubated for 3 days at room temperature in the dark to allow seed germination. After 3 days, selected germinated seeds were examined with a Leica M165FC fluorescent microscope for colonization pattern while other seeds from the same batch were used for colony forming unit (CFU) determination. For the latter, germinated seeds were placed in a 15 ml Falcon tube filled with 10 ml NaCl 0.9% and gently detached by 15 min incubation in a sonication water bath (Memmert WB 14, Germany). Thereafter, the cell suspensions were serially diluted and plated on PIA medium. Colonies were counted after four day incubation at 30°C. To verify statistical significance student's *t*-test was performed. For dual inoculation, colonies were counted under the binocular (to verify green fluorescence, which was not visible by eye on the plate unlike the red color originating from dsRED-tagging). After 7 days of incubation in the Petri dish that contained 1/2 MS medium, new seedlings were harvested and examined for early colonization pattern using a NightOWL LB 983 NC100 (Berthold technologies, Germany).

#### Persistence *in planta*

Seeds of maize and lupin were bacterized using the procedure described above. After 3 days of germination, four seeds for each treatment were transferred to 50 ml Falcon tubes filled with vermiculite (one seed per tube). Plants were transferred to a greenhouse with natural light, approximately 25°C and 70% humidity. 7 days later, a second inoculation step was carried out on these vermiculite microcosms by adding 4 ml of a cell suspension adjusted to an OD_600_ of 0.25 to each Falcon (NaCl for the control microcosms). Plants were fertilized once a week with MIOPLANT fertilizer (Migros, Switzerland) using half the concentration recommended by the manufacturer and watered twice a week. They were harvested after 28 days. To determine CFUs, roots were gently ground in NaCl 0.9% and ground tissues serially diluted and plated on PIA plates (see above, Early colonization).

### Oxalate measurements in plant tissues

Oxalate measurements in lupin and maize root tissues were performed after 3 and 28 days with an enzymatic kit from LIBIOS (France). Prior to analysis, washed roots were weighted and ground in liquid N_2_. The resulting powder was extracted in twice its weight of water for 30 min under continuous shaking. Thereafter, the extract was centrifuged at 13,000 rpm for 5 min and 10 μl of the supernatant was used for oxalate quantification according to the manufacturer's protocol.

### Construction of phylogenetic tree of *Burkholderia* species

Forty one *Burkholderia* 16S rRNA gene sequences were retrieved from the NCBI database (http://www.ncbi.nlm.nih.gov/). 1130 bp long sequences were aligned using ClustalW (Thompson et al., 1994) in MEGA5.05 software (Tamura et al., 2011). Phylogenetic trees were obtained by applying the Neighbor-Joining (NJ) method in the MEGA 5.05 software. The tree topology was inferred with a Kimura 2-parameter correction model (Kimura, 1980) and with 1000 bootstrap replications. 16S rRNA gene sequence of *Ralstonia solanacearum* LMG 2299 was used as an outgroup.

## Results

### Oxalotrophy is widespread in plant-associated *Burkholderia* species but absent from opportunistic pathogenic species

**Figure 1:**
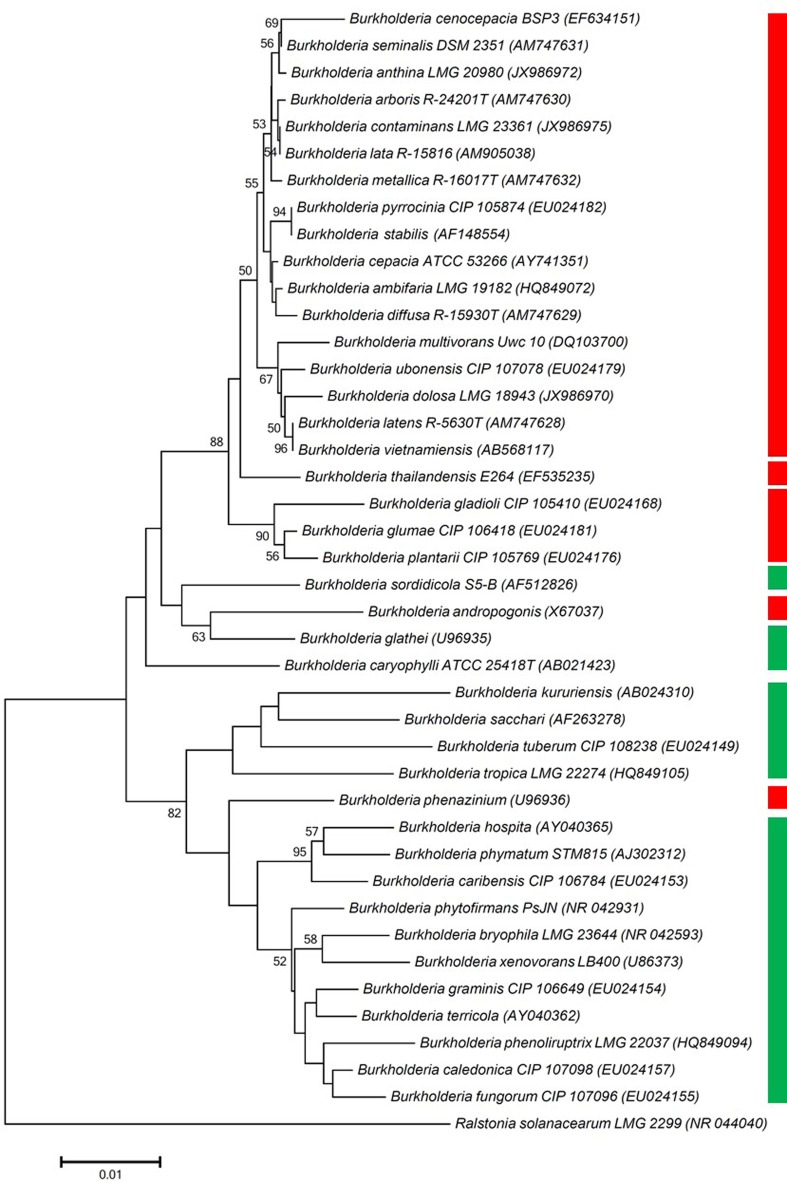
**Phylogenetic tree, constructed using one representative 16S rRNA gene sequence per *Burkholderia* species included in the oxalotrophy assay**. Branches corresponding to partitions reproduced in less than 50% bootstrap replicates are collapsed. Only bootstrap values exceeding 50% are labeled. The percentage of replicate trees in which the associated taxa clustered together in the bootstrap test (1000 replicates) are shown next to the branches. Green bar indicates oxalotrophy of the tested strains of a given species, red bar indicates inability to degrade oxalate (see also Table 1 for detailed results).

Fifty eight strains, which belong to 41 different species were tested for their ability to utilize oxalate as a sole carbon source. None of the strains from the *Burkholderia cepacia* complex species could grow on oxalate (Table 1, Figure 1). Likewise, all plant pathogenic *Burkholderia*, including strains of *B. glumae, B. plantarii*, and *B. gladioli* were unable to do so. In contrast, all *Burkholderia* strains that belonged to the “plant beneficial cluster” (Suarez-Moreno et al., 2012) were oxalotrophic, with the exception of *B. phenazinium*, which could not grow on oxalate (Table 1) and from which the *frc* gene [formyl-CoA transferase, catalyzing the first step of oxalate catabolism (Khammar et al., 2009)] could not be amplified (data not shown). The ability or inability to degrade oxalate was conserved within the same species, as shown for diverse examples (Table 1). The almost universal trait of plant-associated *Burkholderia* to utilize oxalate and the incapacity of all tested plant or human opportunistic pathogens to do so led us to hypothesize that oxalotrophy might be involved in the establishment of mutualistic interactions between bacteria and plants.

**Table 1:** **Oxalate degradation ability (OX) in various species of the *Burkholderia* genus**.

**Species**	**Strain**	**OX**	**Species**	**Strain**	**OX**
**Plant beneficial environ. *Burkhoderia* sp**.			***Burkholderia cepacia* complex sp**.		
*B. caledonica*	LMG19076	+	*B. ambifaria*	LMG17828	−
*B. caribensis*	LMG18531	+	*B. anthina*	LMG21821	−
*B. caryophylli*	LMG2155	+	*B. arboris*	LMG24066	−
*B. bryophila*	LMG23646	+	*B. cenocepacia*	R−6274	−
*B. fungorum*	LMG16225	+	*B. cepacia*	ATCC25416	−
*B. graminis*	LMG18924	+	*B. contaminans*	LMG23361	−
*B. hospita*	LMG20598	+	*B. diffusa*	LMG24065	−
	Isolate NS11	+	*B. dolosa*	LMG18941	−
	Isolate NS7	+	*B. lata*	LMG22485	−
*B. kururiensis*	LMG19447	+	*B. latens*	LMG24064	−
*B. phenoliruptrix*	LMG22037	+	*B. metallica*	LMG24068	−
*B. phymatum*	LMG21445	+	*B. multivorans*	LMG18825	−
*B. phytofirmans*	LMG22487	+	*B. pyrrocinia*	LMG14191	−
*B. sacchari*	LMG19450	+		LMG21822	−
*B. terricola*	FN313521	+		LMG21823	−
	LMG20594	+	*B. seminalis*	LMG24067	−
*B. tropica*	LMG22274	+	*B. stabilis*	Isolate R6270	−
*B. tuberum*	LMG21444	+		LMG14294	−
*B. xenovorans*	LMG21463	+	*B. ubonensis*	LMG20358	−
*B. phenazinium*	LMG2247	−	*B. vietnamiensis*	LMG18835	−
	Isolate S1	−			
	Isolate S7	−	**Plant pathogenic *Burkholderia* sp**.		
	Isolate S18	−	*B. gladioli*	LMG2216	−
	Isolate 1S9	−		LMG11626	−
				LMG18157	−
**Unclassified *Burkholderia* sp**.			*B. glumae*	LMG2196	−
				ATCC33617	−
*B. glathei*	LMG14190	+		AU6208	−
*B. sordidicola*	LMG22029	+		ATCC43733	−
*B. thailandensis*	LMG20219	−	*B. plantarii*	Isolate TT	−
*B. andropogonis*	LMG2129	−		Isolate VV	−
				LMG9035	−

Oxalate degradation for pure Burkholderia cultures was revealed by a halo surrounding growing colonies when inoculated on a minimal medium with calcium oxalate as a sole carbon source (see Materials and Methods for details).

### Oxalotrophy is involved in successful plant colonization by *B. phytofirmans*

**Figure 2:**
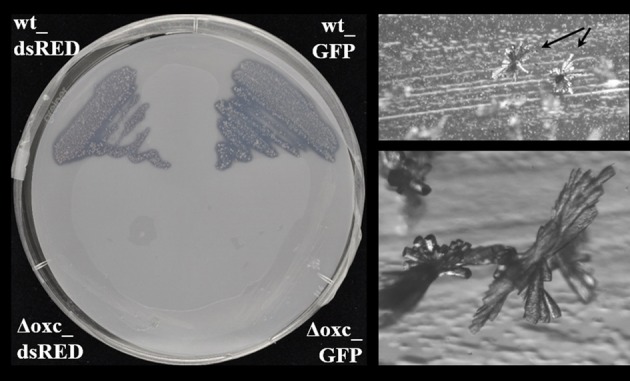
**Assessment of oxalotrophy in the dsRed and GFP-tagged wild-type (wt_dsRed respectively wt_GFP) and the accordingly tagged (Δ*oxc*_dsRED respectively Δ*oxc*_GFP) Δ*oxc* mutants of *B. phytofirmans* (left) strain**. Both tagged wild-type strains (upper half of the Petri dish) showed a cleared halo around the grown colonies, which indicates degradation of the Ca-oxalate present in the upper layer of the minimal medium. Both tagged Δ*oxc* mutant strains (lower half of the Petri dish) were unable to grow on the minimal medium with oxalate as a sole carbon source. Picture was taken after 10 days of growth at room temperature. In the oxalate degrading strains, characteristic crystal structures (most probably CaCO_3_) were formed above the agar surface (right, arrows, and zoomed view below).

To evaluate the role of oxalotrophy in plant colonization, the oxalate decarboxylase gene *oxc* was inactivated in the broad-host endophytic bacterium *B. phytofirmans* PsJN (Sessitsch et al., 2005). The *oxc* gene is the second gene in a putative oxalate catabolism gene cluster, which contains the putative oxalate/formate antiporter Bphyt_6739, *oxc*, and the formyl-coA transferase gene *frc* (Bphyt_6741) (Figure S1). As expected, oxalotrophy was abolished in the mutant strain (Figure 2). The wild-type and the Δ*oxc* mutant were marked with either GFP or dsRED to allow monitoring of their plant colonization abilities (see Materials and Methods for details). The marked strains exhibited the same growth behavior in LB medium in single as well as in mixed inoculation experiments, indicating that the marker genes (GFP, dsRED) did not affect the results (Figure S2).

**Figure 3:**
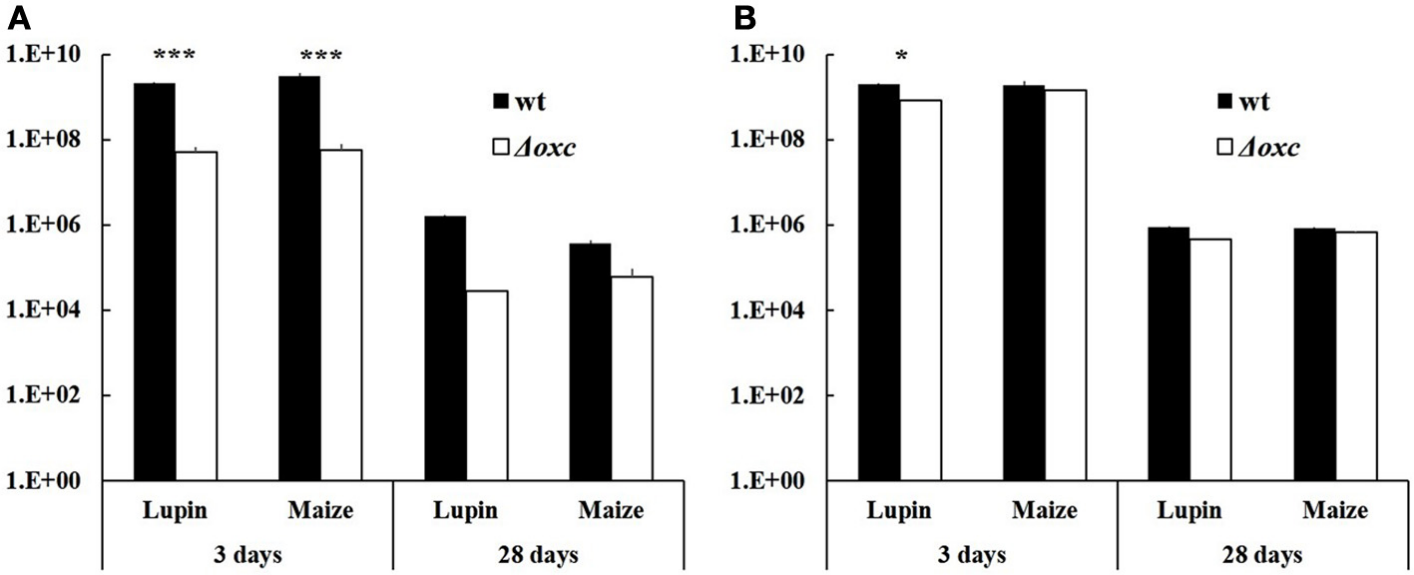
**Abundance of *B. phytofirmans* PsJN wild-type and Δ*oxc* mutant after seed germination (3 days) and after one-month microcosm cultivation in vermiculite (28 days)**. Seeds were either bacterized with one strain (single inoculation, **A**) or with a 1:1 mixture of wt and Δ*oxc* mutant (dual inoculation, **B**). Results are expressed as CFU per seed after 3 days and as CFU per g root fresh weight after 28 days. Average of 2–3 replicates (seeds/plants) are shown, with the exception of one case, where no mutant cells were retrieved in two out of three replicate plants (**A**, lupin, 28 days, Δ*oxc*). Stars indicate significant differences between wild-type and mutant (Student's *t*-test, *n* = 2–4, ^*^P < 0.05, ^***^P < 0.001).

**Figure 4:**
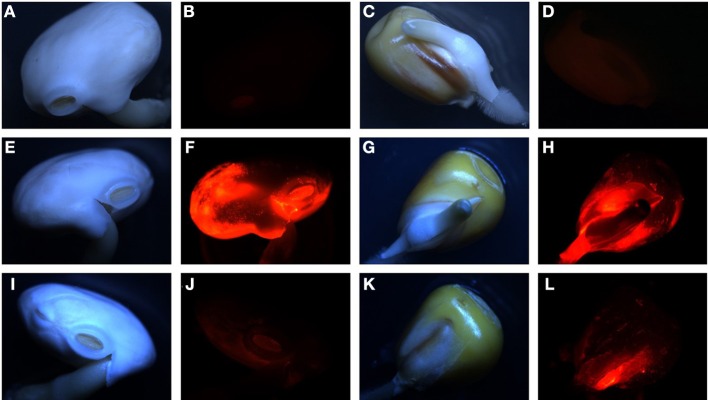
**Representative pictures of seed colonization of lupin (A,B,E,F,I,J) and maize (C,D,G,H,K,L) by dsRED-tagged wild-type (E–H) or Δ*oxc* mutant (I–L) after 3 days. A-D:** non inoculated seeds. Pictures were taken using a Leica M165FC fluorescent microscope, under normal light **(A,C,E,G,I,K)** or dsRED fluorescent filter **(B,D,F,H,J,L)** with 0.4 s. exposure in all cases.

**Figure 5:**
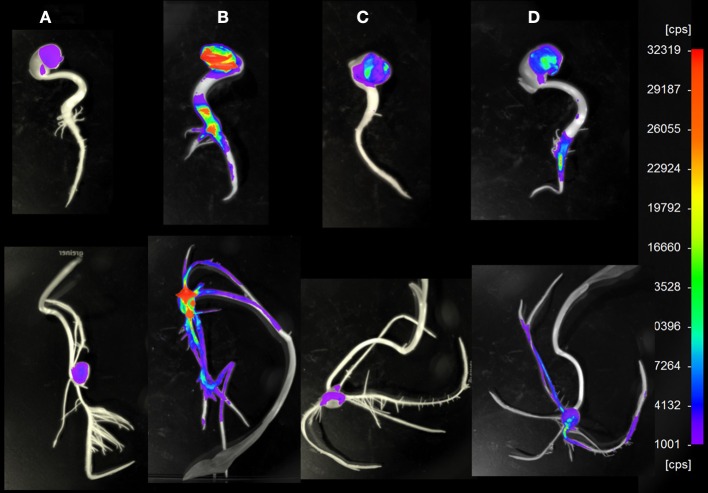
**Representative pictures of lupin (upper row) and maize (lower row) 7 day old seedlings colonized by *B. phytofirmans* PsJN wild-type or Δ*oxc* mutant in single or combined inoculation**. For imaging, a NightOWL LB 983 NC100 was used, under conditions where dsRED-tagged cells are visible. **(A)**: non inoculated control, **(B)**: inoculated with dsRED-tagged wild-type, **(C)**: inoculated with dsRED-tagged Δ*oxc*, **(D)**: inoculated with GFP-tagged wild-type and dsRED-tagged Δ*oxc* mutant. cps: counts per second.

Sterilized seeds of lupin and maize were inoculated with (i) the wild-type, (ii) the Δ*oxc* mutant, and (iii) both strains in equal cell densities (approximately 10^7^ cells/ml of inoculation solution). For single inoculation studies, the dsRED-tagged strains were used, as the signal was brighter than in the GFP-tagged strains. For dual inoculations, both combinations were used (GFP-tagged wild-type and dsRED-tagged Δ*oxc*, or dsRED-tagged wild-type and GFP-tagged Δ*oxc*) to avoid any bias due to fluorescent marker genes. When inoculated as single strains, a significant decrease in root colonization capacity was observed in the mutant relative to the wild-type on both lupin and maize (Figure 3A). This difference, which was confirmed by microscopic inspection (Figure 4), was more pronounced at early stages of colonization than after one month of cultivation, especially for maize. In lupin, about a million cells/g root fresh weight could be detected for the wild-type in all three plants after 28 days, yet the mutant was only detectable in one of three plants and present at a much lower population density (100-fold decreased relative to the wild-type). In maize, the difference was less pronounced after one month of cultivation when compared to the beginning of colonization (just below significance level, *P* = 0.055). This difference might be explained by the fact that lupins produced much more oxalate than maize (30 nmol vs. 6 nmol per g root fresh weight after 3 days and 60 nmol vs. 30 nmol after 28 days). When inoculated together with the wild-type, the colonization defect of the Δ*oxc* mutant was restored (Figure 3B), that was confirmed by visual inspection of 7 day-old seedlings (Figure 5). While the Δ*oxc* mutant was not able to spread from the seeds to the roots when inoculated as a pure culture (Figure 5C), this phenotype was partially rescued in the presence of the wild-type strain (Figure 5D).

## Discussion

One major source of oxalate in natural ecosystems is fungal production, e.g., in wood-rotting fungi, where it is involved in lignin degradation or in some phytopathogenic fungi, e.g., *Sclerotinia* or *Botrytis* species, where it acts as virulence factor (Dutton and Evans, 1996; Criscitiello et al., 2013; Heller and Witt-Geiges, 2013). High quantities of oxalate are toxic to animals and humans, due to the formation of calcium- or magnesium oxalate crystals, which can lead to depletion in essential cations or to kidney stone formation (Coe et al., 2010). However, oxalate is also an important metabolite of many plant species, where it is thought to be important for calcium storage and for repelling herbivores (Franceschi and Nakata, 2005). Moreover, oxalate secretion is involved in tolerance to heavy metals including aluminum, as demonstrated e.g., in buckwheat (Klug and Horst, 2010) or in rice (Yang et al., 2000).

When plants grow in situations where nutrients such as phosphate or iron are limited, or when heavy metals are abundant, excretion of organic acids is increased (Meyer et al., 2010). This enhanced secretion of citrate, malate or oxalate enriches the rhizosphere in organic carbon, which can be used by certain microorganisms as a nutritional source. Consequently, those members of the community that possess the metabolic means to catabolize those exudates will be enriched. In a previous study, we observed an overrepresentation of *Burkholderia* species in various development stages of white lupin cluster roots (Weisskopf et al., 2011). This enrichment might be linked to the acidic environment that prevails around mature cluster roots and to the preference of *Burkholderia* species to exist in acidic soils (Stopnisek et al., 2013). Given that most of the *Burkholderia* strains isolated from white lupin were able to utilize oxalate as a carbon source, we asked whether this property is, like acid tolerance, a genus-wide property or is restricted to species that are predominantly associated with plants and/or fungi. By testing strains that belong to 41 different species, we observed that the ability to grow on oxalate as a sole carbon source is restricted to members of the plant-beneficial environmental cluster (Suarez-Moreno et al., 2012) (Figure 1) and absent in pathogenic species, including the human pathogen *B. pseudomallei*, plant pathogens such as *B. plantari* or *B. glumae* and opportunistic pathogens, which belong to the Bcc cluster. Interestingly, virulent strains of *B. glumae*, an important pathogen of rice, have been shown to produce oxalate, while non-virulent ones were not oxalogenic (Li et al., 1999), suggesting that oxalate production might be important for virulence, as it is the case with fungal pathogens. Beyond its role as a virulence factor, oxalate has been postulated to be a common good of pathogenic *Burkholderia* species, including *B. glumae, B. pseudomallei and B. thailandensis* (Goo et al., 2012). Oxalate production in these species is controlled by quorum-sensing and was shown to neutralize the alkalinization of the medium caused by the emission of NH_3_ in the late stationary phase, thereby ensuring that the pH remains at a physiological level (Goo et al., 2012).

Oxalate degradation by plant-beneficial *Burkholderia* might be considered a plant-protecting feature, as lowering the oxalate levels on plant surfaces might alleviate the infection potential of oxalate-producing phytopathogenic fungi or bacteria. This was shown in the case of *Cupriavidus campinensis*, which could significantly reduce disease symptoms caused by the oxalogenic fungi *Botrytis cinerea* or *Sclerotinia sclerotiorum* on *Arabidopsis*, grapevine and tomato plants, while a mutant strain impaired in oxalate degradation showed only reduced protecting potential (Schoonbeek et al., 2007). In order to investigate whether oxalate degradation might provide an advantage in plant colonization, a mutant, in which oxalotrophy is abolished, was generated (Figure 2). The broad-host endophyte *B. phytofirmans* PsJN (Sessitsch et al., 2005) served as a model organism in this study. The colonization behavior of the wild-type and the mutant on plants with moderate (white lupin) or low (maize) oxalate secretion was compared. When inoculated alone, the mutant suffered a drastic disadvantage both in early colonization steps (3 days) and in persistence on the plants (Figures 3A, 4, 5). Similar differences between the wild-type and the mutant were observed for lupin and maize at the early stage of colonization; however, after one month of cultivation the effects were much more dramatic on lupins, where only in one out of three plants mutant cells could be recovered, than on maize, for which the difference between wild-type and mutant was not significant. Surprisingly, when the mutant and the wild-type were inoculated in a 1:1 ratio, the mutant recovered most of its lost capacity to colonize the plants (Figures 3B, 5). This suggests that oxalate might act as a toxic compound for the strains that cannot degrade it. The presence of the wild-type would then alleviate this toxic effect by lowering the levels of free oxalate through oxalotrophy. When grown in glucose-supplemented minimal medium, the mutant's growth was only very marginally reduced upon addition of oxalate, which indicates that oxalate is not toxic under laboratory conditions. However, this does not exclude a putative toxicity of oxalate in the seed or plant environment. Moreover, the better colonization performance of the mutant when co-inoculated with the wild-type might also be explained by the utilization of degradation products resulting from oxalate catabolism of the wild-type.

Roots are the entry point for most endophytic bacteria, which then can spread to above-ground plant tissues. Understanding how plants select for beneficial root and shoot inhabitants and/or against plant pathogenic species is obviously very important for plant health. This work sheds light on the so far overlooked role of oxalotrophy in root colonization, which in the case of *Burkholderia* species selects for plant beneficial bacteria over colonization by plant and even animal pathogens.

## Author contributions

Laure Weisskopf designed the research, Thomas Kost, Kirsty Agnoli and Laure Weisskopf performed experiments, Thomas Kost, Nejc Stopnisek, Kirsty Agnoli and Laure Weisskopf analyzed the data, Laure Weisskopf wrote the MS with help from Kirsty Agnoli, Nejc Stopnisek, Thomas Kost, and Leo Eberl.

### Conflict of interest statement

The authors declare that the research was conducted in the absence of any commercial or financial relationships that could be construed as a potential conflict of interest.

## Supplementary material

Table S1 **Strains and plasmids used in this study**.

Figure S1 **Structure of the gene cluster involved in oxalate degradation and construction of a Δ*oxc* mutant in *B. phytofirmans* PsJN**.

Figure S2 **Growth curves and *in vitro* competition experiment of *B. phytofirmans* PsJN wt and Δ*oxc* mutant**.
