# Supplementary material for: Oxalotrophy, a widespread trait of plant-associated Burkholderia species, is involved in successful root colonization of lupin and maize by Burkholderia phytofirmans
Source: Front Microbiol. 2014 Jan 9;4:421. doi: 10.3389/fmicb.2013.00421 (PMC3886118; doi:10.3389/fmicb.2013.00421)
Supplement: Table S1 — Strains and plasmids used in this study. [file DataSheet1.PDF]

Table S1 Strains and plasmids used in this study. References are listed for the strains without ATCC or LMG number. CF: cystic fibrosis; wt: wild-type; Δ*oxc*: mutant strain with interrupted oxalate decarboxylase gene; GFP: green fluorescent protein; dsRED: red fluorescent protein.

**Species**	**Strain number**	**Strain origin**	**Reference**
B. ambifaria	LMG17828	roots	
B. andropogonis	LMG2129	*Sorghum bicolor*	
B. anthina	LMG21821	CF patient	
B. arboris	LMG24066	soil	
B. bryophila	LMG23646	moss	
B. caledonica	LMG19076	soil rhizosphere	
B. caribensis	LMG18531	soil	
B. caryophylli	LMG2155	*Dianthus caryophyllus*	
B. cenocepacia	R-6274	CF patient	Gotschlich et al., 2001
B. cepacia	ATCC25416	*Allium cepa*	
B. contaminans	LMG23361	sheep milk	
B. diffusa	LMG24065	CF patient	
B. dolosa	LMG18941	CF patient	
B. fungorum	LMG16225	*Phanerochaete chrysosporium*	
B. gladioli	LMG2216	*Gladiolus* sp.	
B. gladioli	LMG18157	CF patient	
B. gladioli	LMG11626	poisoned bongkrek	
B. glathei	LMG14190	soil	
B. glumae	LMG2196	*Oryza sativa*	
B. glumae	AU6208	clinical isolate	
B. glumae	ATCC33617	*Oryza sativa*	
B. graminis	LMG18924	roots	
B. hospita	NS7	soil	this study
B. hospita	NS11	soil	this study
B. hospita	LMG20598	soil	
B. kururiensis	LMG19447	water	
B. lata	LMG22485	soil	
B. latens	LMG24064	CF patient	
B. metallica	LMG24068	clinical isolate	
B. multivorans	LMG18825	CF patient	
B. phenazinium	S1	moss	Opelt and Berg, 2004
B. phenazinium	S7	moss	Opelt and Berg, 2004
B. phenazinium	S18	moss	Opelt and Berg, 2004

B. phenazinium	1S9	moss	Opelt and Berg, 2004
B. phenazinium	LMG2247	soil	
B. phenoliruptrix	LMG22037	chemostat	
B. phymatum	LMG21445	root nodules	
B. phytofirmans	LMG22487 (PsJN)	onion roots	
B. phytofirmans Δ*oxc* (pin62)	LMG22487	dsRED-tagged wild-type PsJN	this study
B. phytofirmans (pBBR1MCS-2-gfpmut3-1)	LMG22487	GPF-tagged wild-type of PsJN	this study
B. phytofirmans Δ*oxc*	LMG22487	Δ*oxc* mutant of PsJN	this study
B. phytofirmans (pin62)	LMG22487	dsRED-tagged Δoxc mutant of PsJN	this study
B. phytofirmans Δ*oxc* (pBBR1MCS-2-gfpmut3-1)	LMG22487	GPF-tagged Δoxc of PsJN	this study
B. plantarii	ATCC43733	*Oryza sativa*	
B. plantarii	ATCC43733TT	*Oryza sativa*	
B. plantarii	ATCC43733VV	*Oryza sativa*	
B. plantarii	LMG9035	*Oryza sativa*	
B. pyrrocinia	LMG21822	soil	
B. pyrrocinia	LMG14191	soil	
B. pyrrocinia	LMG21823	water	
B. sacchari	LMG19450	soil	
B. seminalis	LMG24067	clinical isolate	
B. sordidicola	LMG22029	*Phanerochaete sordida*	
B. stabilis	R-6270	CF patient	Gotschlich et al., 2001
B. stabilis	LMG14294	CF patient	
B. terricola	FN313521	rhizosphere	Gasser et al., 2009
B. terricola	LMG20594	soil	
B. thailandensis	LMG20219	soil	
B. tropica	LMG22274	roots	
B. tuberum	LMG21444	root nodules	
B. ubonensis	LMG20358	soil	
B. vietnamiensis	LMG18835	CF patient	
B. xenovorans	LMG21463	soil	
Escherichia coli	DH10B	Invitrogen Top10 cells	Invitrogen

Escherichia coli	MM294 (pRK2013)		Figurski and Helinski, 1979; Nakagawa et al., 1996
Escherichia coli	DH5α	Invitrogen DH5α cells	Invitrogen
Escherichia coli	MT102 (pSB403)		Huber et al., 2003
Escherichia coli	CC118λpir		Herrero et al., 1990

**Plasmid**	**Specification**	**Characteristics**	**Reference**
pin62	DsRed donor plasmid	oripBBR mob+, Cmr, DSRed	Vergunst et al., 2010
pBBR1MCS-2-gfpmut3-1	GFP donor plasmid	pBAH7, KmR, GFP	Rothballer et al., 2005
pSHAFT2	Broad-host-range suicide plasmid	Cmr, mobilisable for conjugation	S. Shastri and M.S.Thomas, manuscript in preparation

Figure S1 (A) Structure of the gene cluster involved in oxalate degradation in *B. phytofirmans* PsJN (source: http://www.ncbi.nlm.nih.gov). Genes are located on chromosome 2. Bphyt_6739: putative oxalate transporter; Bphyt_6740: oxalate decarboxylase; Bphyt_6741: formyl CoA transferase. (B) Construction of a Δ*oxc* mutant in *B. phytofirmans* PsJN. The diagram illustrates the cloning steps involved in the mutagenesis of the *oxc* gene.

A

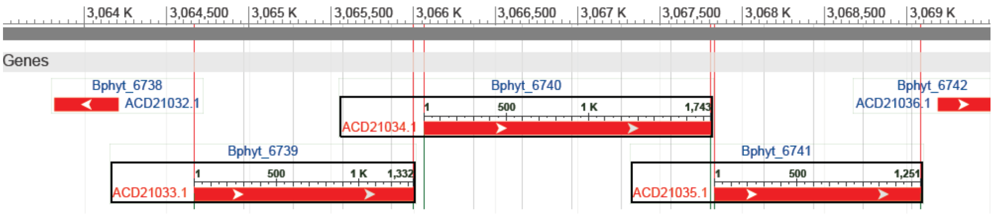

B

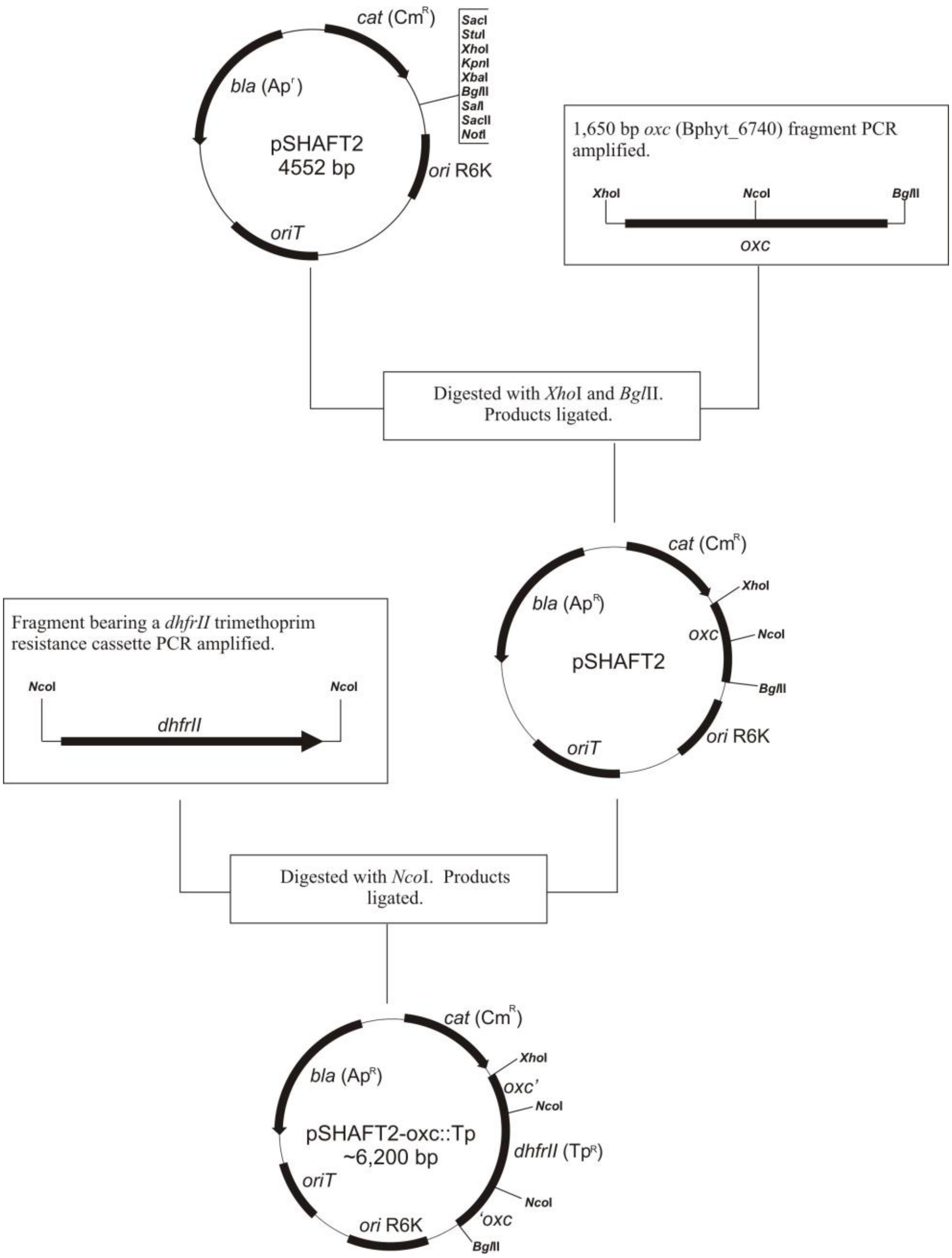

Figure S2 Growth curves and *in vitro* competition experiment of *B. phytofirmans* PsJN wild-type strain and Δ*oxc* mutant. (A) Optical densities of each strain grown as pure culture in LB. (B) Colony forming units (CFU) / ml of GFP-tagged wild-type and dsRED-tagged Δ*oxc* mutant when grown together. CFU/ml of dsRED-tagged wild-type and GFP-tagged Δ*oxc* mutant in mixed culture.

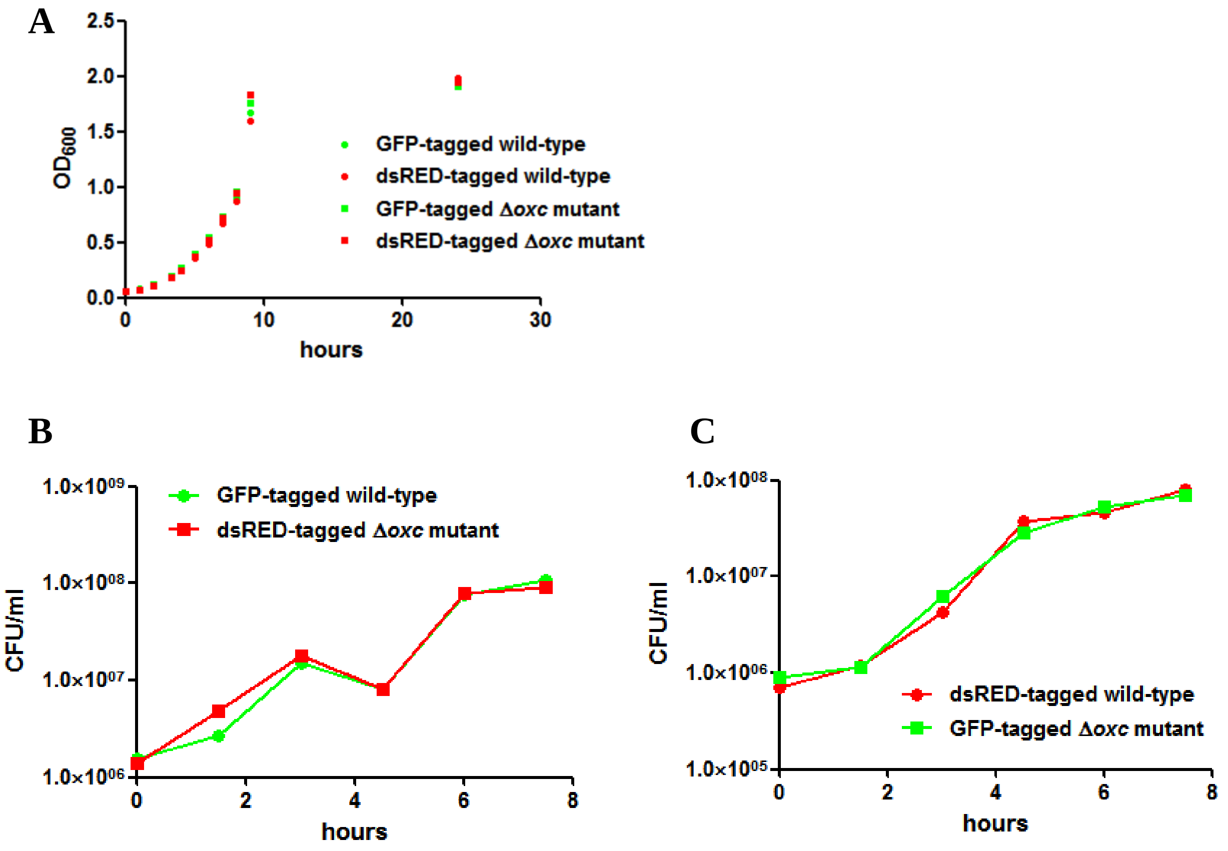

References

Figurski, D.H., and Helinski, D.R. (1979). Replication of an origin-containing derivative of plasmid RK2 dependent on a plasmid function provided in trans. *Proc. Nat. Acad. Sci.* 76**,** 1648-1652.

Gasser, I., Müller, H., and Berg, G. (2009). Ecology and characterization of polyhydroxyalkanoate-producing microorganisms on and in plants. *FEMS Microbiol. Ecol.* 70**,** 142-150. doi: 10.1111/j.1574-6941.2009.00734.x.

Gotschlich, A., Huber, B., Geisenberger, O., Tögl, A., Steidle, A., Riedel, K., Hill, P., Tümmler, B., Vandamme, P., Middleton, B., Camara, M., Williams, P., Hardman, A., and Eberl, L. (2001). Synthesis of Multiple N-Acylhomoserine Lactones is Wide-spread Among the Members of the Burkholderia cepacia Complex. *Syst. Appl. Microbiol.* 24**,** 1-14. doi: 10.1078/0723-2020-00013.

Herrero, M., De Lorenzo, V., and Timmis, K.N. (1990). Transposon vectors containing non-antibiotic resistance selection markers for cloning and stable chromosomal insertion of foreign genes in gram-negative bacteria. *J. Bacteriol.* 172**,** 6557-6567.

Huber, B., Eberl, L., Feucht, W., and Polster, J. (2003). Influence of polyphenols on bacterial biofilm formation and quorum-sensing. *Z. Naturforsch. C* 58**,** 879-884.

Nakagawa, S., Ishino, S., and Teshiba, S. (1996). Construction of Catalase Deficient Escherichia coli Strains for the Production of Uricase. *Biosc. Biotech. Biochem.* 60**,** 415-420. doi: 10.1271/bbb.60.415.

Opelt, K., and Berg, G. (2004). Diversity and Antagonistic Potential of Bacteria Associated with Bryophytes from Nutrient-Poor Habitats of the Baltic Sea Coast. *Appl. Environ. Microb.* 70**,** 6569-6579. doi: 10.1128/aem.70.11.6569-6579.2004.

Rothballer, M., Schmid, M., Fekete, A., and Hartmann, A. (2005). Comparative *in situ* analysis of ipdC–gfpmut3 promoter fusions of *Azospirillum brasilense* strains Sp7 and Sp245. *Environ. Microbiol.* 7**,** 1839-1846. doi: 10.1111/j.1462-2920.2005.00848.x.

Vergunst, A.C., Meijer, A.H., Renshaw, S.A., and O'callaghan, D. (2010). *Burkholderia cenocepacia* Creates an Intramacrophage Replication Niche in Zebrafish Embryos, Followed by Bacterial Dissemination and Establishment of Systemic Infection. *Infect. Immun.* 78**,** 1495-1508. doi: 10.1128/iai.00743-09.
